# Multisite Musculoskeletal Pain Is Associated with Long-Term Declined Physical Quality of Life and Knee-Related Quality of Life in Older Adults with or at Risk of Knee Osteoarthritis

**DOI:** 10.3390/medicina60081305

**Published:** 2024-08-12

**Authors:** Bader A. Alqahtani, Aqeel M. Alenazi

**Affiliations:** Department of Health and Rehabilitation Sciences, Prince Sattam Bin Abdulaziz University, Al-Kharj 11942, Saudi Arabia; aqeel.alanazi@psau.edu.sa

**Keywords:** multisite musculoskeletal pain, health-related quality of life, older adults, knee osteoarthritis

## Abstract

*Background and Objectives*: This study aimed to examine the longitudinal impact of multisite musculoskeletal pain on physical and mental health-related quality of life among individuals with or at risk of knee osteoarthritis. *Materials and Methods*: This study is a prospective longitudinal design over 8 years of follow-up. Data from 4796 participants aged between 45 and 79 years were acquired from the Osteoarthritis Initiative. Based on self-reported physician-diagnosed osteoarthritis and grade ≥2 in either knee using Kellgren and Lawrence grade at baseline, individuals at risk were classified as those who did not have knee osteoarthritis at baseline but could develop osteoarthritis throughout the study. Physical and mental components of health-related quality were assessed over an 8-year follow-up period using both knee injury and osteoarthritis outcome scores and the 12-item Short-Form Health Survey. Multisite pain was examined using a self-reported questionnaire for 20 sites. Two separate generalized estimating equations modeled with a linear regression analysis were utilized. *Results*: The results showed that participants with one painful site (Beta [B] = −0.92, *p* = 0.01), two painful sites (B = −1.94, *p* < 0.001), and multisite pain (≥3 painful sites) (B = −4.68, *p* < 0.001) were significantly associated with declined physical health-related quality of life over time when compared to those with no painful site at baseline after adjustments for covariates. However, there was no significant association with declined mental health-related quality of life over time. *Conclusions*: This study revealed that baseline multisite musculoskeletal pain was linked to declining physical and knee injury and osteoarthritis outcome score quality of life among individuals with or at risk of knee osteoarthritis. Moreover, having baseline multisite pain and two painful sites were associated with a decline in physical and knee injury and osteoarthritis outcome score quality of life, while mental health-related quality of life did not show a significant association with multisite pain. Therefore, it is imperative for primary healthcare settings to prioritize the assessment of multisite musculoskeletal pain and develop interventions aimed at preserving and enhancing physical health-related quality of life in people with or at risk of osteoarthritis.

## 1. Introduction

Musculoskeletal disorders are one of the most prevalent conditions in the United States; according to the 2013–2015 National Health Interview Survey, one out of every two adults in the United States has musculoskeletal disorders. Chronic pain and function loss are the primary mechanisms by which musculoskeletal disorders induce disability and lower quality of life [1]. In addition, multisite musculoskeletal pain (MMP), which affects more than one anatomical site, is very common and more often reported than single-site pain [2,3]. Previous studies have reported that multisite pain is associated with reduced upper and lower extremity mobility, decreased balance, increased fall risk, disability, poor physical function, depressive symptoms, and anxiety [4,5,6,7]. Notably, MMP is more prevalent among older adults and women [8]. Thus, MMP poses a substantial burden to the healthcare system.

More specifically, among people who have or are at risk of osteoarthritis (OA), pain involving three or more locations is associated with the complexity of chronic pain. MMP indicates increased symptom severity and a greater negative effect on daily functioning and quality of life. MMP has also been associated with reduced health-related quality of life (HRQOL) in older adults and the general population [9,10]. Exploring this association in different subgroups, such as people with or at risk of knee osteoarthritis (OA), will enhance our understanding and improve our clinical practice. However, studies on the longitudinal association between MMP and HRQOL in individuals with knee OA or at risk of knee OA are lacking. Previous studies have assessed the association between multisite pain and mental and physical HRQOL; however, these studies were primarily cross-sectional in design, which inherently limits the assessment of long-term impact and causality relationship and often overlooks the dimensions of pain on either the right or left side [11,12]. By following participants over the years, we will provide critical insights into how MMP contributes to the progressive decline in physical quality of life and knee-related quality of life (KOOS QOL). Moreover, previous research has often assessed multisite pain using a limited number of pain sites [13,14]. Thus, the results may have provided a narrow perspective on the complex and multifaceted nature of pain experiences in individuals with knee OA. In addition, previous studies used more generic QOL questionnaires, whereas using more specific knee-related QOL questionnaires like the knee injury and osteoarthritis outcome score (KOOS) would provide more specific aspects and cover both the short- and long-term consequences of an injury of the knee in individuals with or at risk of knee OA [12,13,15]. 

Therefore, in this study, we aimed to examine the longitudinal impact of MMP on mental and physical HRQOL, and knee-specific HRQOL using the KOOS among individuals with or at risk of knee OA over 96 months of follow-up. 

## 2. Methods

### 2.1. Study Design and Cohort 

We used longitudinal data from a prospective cohort, namely the Osteoarthritis Initiative (OAI). A full description of the OAI study is available at https://nda.nih.gov/oai/ (accessed on 1 July 2024). Briefly, the OAI is a multisite longitudinal study conducted in the United States with the primary objective of understanding OA onset and progression and developing evidence-based strategies for the prevention and treatment of knee OA. The OAI study involved 4796 participants aged 45–79 years at the time of recruitment who had or were at high risk of symptomatic knee OA. Individuals at risk were defined as those who did not have knee OA at baseline but could develop OA throughout the study. All the participants in the main study completed eight visits, including biospecimen collection, imaging, and clinical assessments, with variable time intervals between visits from baseline to 96 months. 

All procedures in the OAI that involved human participants adhered to the ethical guidelines of the institutional and/or national research committee and complied with the 1964 Helsinki Declaration and its later amendments or comparable ethical standards. Each participant provided informed consent before enrollment in the study. This study was approved by the Institutional Review Board of the University of California, San Francisco, and its affiliates (approval number: FWA00000068). Institutional Review Board approval was also obtained from all four clinical sites located at Brown University in Rhode Island; Ohio State University, Columbus, Ohio; University of Maryland/Johns Hopkins University Joint Center in Baltimore, Maryland; and University of Pittsburgh in Pennsylvania. 

For the present study, all participants of the OAI study were included in the analysis.

### 2.2. Outcome Measures

The primary outcome was HRQOL, measured using the Medical Outcomes Study Short-Form 12 (SF-12) at various time points over seven visits across a period of up to 96 months [16]. The SF-12 is a self-report questionnaire comprising 12 items across eight health domains, generating composite scores for physical component score (PCS) and mental component score (MCS). The scores were standardized to a mean of 50 and a standard deviation of 10 in the general US population; deviations from the score of 50 indicate better or worse performance in PCS and MCS [16]. The SF-12 is a validated and reliable tool used widely in populations with OA [16,17]. 

We also used the knee injury and osteoarthritis outcome score (KOOS) for quality of life, which includes 42 items and is a self-reported measure of knee outcomes. The KOOS covers five subscales: pain, symptoms, activities of daily living function, sport and recreation function, and knee-related quality of life [15]. In the present study, the KOOS for quality of life subscale was used to examine the participants’ knee-related quality of life. This subscale comprises four items and has been normalized to a 0–100 score, with a higher score indicating better health status. 

### 2.3. Multisite Pain

Multisite pain for a total of 20 sites was examined using a self-reported questionnaire. The questionnaire comprised questions related to pain in the shoulder (right/left), elbow (right/left), wrist (right/left), hands/fingers (right/left), ankle (right/left), foot (right/left), knee (right/left), hip (right/left), back, neck, temporomandibular joint (TMJ), and other joint. Examples of the questions are as follows: “Do you have right shoulder pain, aching, or stiffness for more than half the days in the past 30 days?”, “Do you have right knee pain, aching, or stiffness in the past 12 months?”, and “Do you have pain or aching in the TMJ/jaw joint/in front of the ear in the past 6 months?”. Baseline self-reported painful sites were categorized as yes/no for each site. Based on this questionnaire, multisite pain was categorized into four groups: no painful site, one painful site, two painful sites, and multisite pain (three or more painful sites).

### 2.4. Covariates

Demographic characteristics (age, sex, race, education, and body mass index [BMI]), other clinical variables (depressive symptoms, physical activity level, and Kallgren–Lawrence [KL] scores for the right and left knees), and multiple long-term chronic conditions (MLTCs) were considered covariates [18]. Age was recorded in years as a continuous variable. The race variable was dichotomized into White and other (such as Black/Asian/other non-White) because most of the included participants were White. Education level was dichotomized into high school/lower and college/graduate. BMI was calculated by dividing body mass (kg) by the square of height (m^2^). The Center for Epidemiologic Studies—Depression Scale measured using a self-reported 20-item questionnaire was used for depressive symptoms [19]. Physical Activity in the Elderly Scale was used to measure physical activity levels [20]. KL scores for the right and left knees were measured at baseline, with grades of 0–4 to measure knee OA severity; higher grades indicate greater severity. MLTCs were derived from the Charlson comorbidity index and classified into three distinct categories: no chronic condition, one chronic condition, and two or more chronic conditions.

### 2.5. Data Analysis

Data were analyzed based on the types of variables, either continuous or categorical, for descriptive statistics related to baseline demographics and clinical variables. The data were analyzed using a chi-square test and one-way ANOVA for categorical and continuous variables, respectively, to identify differences among the MLTC categories (none, 1, or ≥2 diseases).

To examine whether multisite pain affects the PCS and MCS of HRQOL and the KOOS for quality of life, three separate generalized estimating equations (GEEs) modeled with a linear regression analysis were utilized. The models were related to the PCS and MCS of HRQOL and the KOOS for quality of life each. Multisite pain categories were set as predictor variables, with no painful sites as the reference category. The outcome variables were the PCS and MCS of HRQOL and the KOOS for quality of life. All models were adjusted for age, sex, race, educational level, BMI, depressive symptoms, physical activity, and KL scored for the right and left knee and MLTCs. An alpha level was set at 0.05 for all analyses, and all analyses were performed using IBM SPSS for Mac version 25.0 (SPSS Inc. Chicago, IL, USA).

## 3. Results

A total of 4795 participants with or at risk of knee OA were included in the current analysis. Table 1 shows the baseline demographic and clinical variables. Based on the self-reported painful sites across the 20 sites, the participants were classified into no painful site (*n* = 198), one painful site (*n* = 418), two painful sites (*n* = 672), and multisite pain (*n* = 3507) groups.

The prevalence of having multisite pain was 73.1%, whereas the prevalence of having no painful sites was 4.1%. Multisite MSK pain, sex, race, BMI, education, depressive symptoms, physical activity level, KL grades for right and left knees, MLTCs, PCS of HRQOL, MCS of HRQOL, and the KOOS for quality of life were significantly different among the multisite pain groups.

Longitudinal analysis using GEE linear regression for the association between multisite pain and PCS of HRQOL showed that participants with one painful site (Beta [B] = −0.92, *p* = 0.01), two painful sites (B = −1.94, *p* < 0.001), and multisite pain (B = −4.68, *p* < 0.001) were significantly associated with decreased PCS of HRQOL over time compared with those with no painful site at baseline (Table 2).

Furthermore, longitudinal analysis using GEE linear regression for the association between multisite pain and MCS of HRQOL showed that participants with one painful site (Beta [B] = −0.20, *p* = 0.46), two painful sites (B = 0.06, *p* = 0.79), and multisite pain (B = 0.21, *p* = 0.36) were not significantly associated with a decline in the MCS of HRQOL over time compared with those with no painful site at baseline (Table 3).

Finally, longitudinal analysis of the association between multisite pain and the KOOS for quality of life showed that participants with one painful site (Beta [B] = −6.97, *p* < 0.001), two painful sites (B= −9.96, *p* < 0.001), and multisite pain (B = −17.56, *p* < 0.001) were significantly associated with a decline in the KOOS for quality of life over time compared with those with no painful site at baseline (Table 4).

## 4. Discussion

This study examined the association between baseline multisite pain and longitudinal decline in physical and mental HRQOL among individuals with or at risk of knee OA. We found that baseline multisite pain and two painful sites were associated with a decline in the PCS of HRQOL and the KOOS for quality of life. Having one painful site was also associated with a longitudinal decline in the KOOS for quality of life subscale scores. However, the MCS of HRQOL was not associated with multisite pain.

The results of the present study were partially consistent with those of previous studies that examined multisite pain and quality of life. A previous study found that multisite pain was associated with poor physical and mental quality of life in adults aged 45 years and older [13]. This result was consistent with our findings on the PCS of HRQOL but not on the MCS of HRQOL. However, it should be noted that this previous study had a cross-sectional design, and our baseline analysis showed similar results regarding poor mental quality of life in the multisite pain groups. Another study examined the association between multisite pain and HRQOL among older adults in the UK [12]. This study found that with every additional pain site, there was a decrease in PCS and MCS of HRQOL in this population. The results were partially consistent with our results in terms of decreased PCS but in contrast with MCS as our study did not find a significant association between multisite pain and MCS of HRQOL. These contradictory results could be attributed to a different design (a cross-sectional design), including only older adults, and a different classification of multisite pain than our study. (i.e., 1–3 sites, 4–6 sites, 7–11 sites, and 12–44 sites).

Notably, research on the association between multisite pain and quality of life in patients with knee OA is limited. Hoogeboom et al. used a similar dataset, the OAI study, and found similar results after 4 years of follow-up [21]. However, this study only used the KOOS for quality of life and had limited time points. Our study used a longer follow-up time point of seven times from baseline to 96 months of follow-up. Additionally, we used general quality of life scales, including the PCS and MCS of HRQOL using the SF-12, to comprehensively highlight the impact of multisite pain and quality of life. Further research is needed to examine the clusters and classification of sites on the quality of life of patients with OA. A previous work from China examined the associated factors with HRQOL [22].This study found that pain in places other than the knee was significantly associated with decreased MCS of HRQOL, in contrast to our findings. This could be attributed to the cross-sectional design and different classification for multisite pain as it was recorded as yes or no without counting the number of painful sites.

Although multisite pain is common in individuals with or at risk of OA, most studies have ignored the impact of multisite pain in favor of focusing on single-site pain. As a result, our research on the characteristics and associations between multisite pain and HRQOL in individuals with or at risk of OA could provide essential information regarding the state of health and potential variables linked to quality of life. Moreover, our findings illustrated a reduction in the PCS of HRQOL as MMP increased. This suggests that the MMP can serve as a valuable quantifiable metric and a focal point for interventions designed to preserve and improve the PCS of HRQOL in individuals with or at risk of OA.

The present study has several strengths, such as the longitudinal design and large sample size, which helped to understand the impact of MMP on HRQOL among adults with or at risk of OA.

Nevertheless, this study also had some limitations. An important limitation of this study was the covariates that prevented us from controlling for various covariates. For instance, although the one-way ANOVA showed a significant decline in the MCS of HRQOL at baseline when comparing MMP to no multisite pain, the longitudinal results were not significant. This may indicate that after controlling for covariates, the MCS of HRQOL was no longer significant. Moreover, the HRQOL assessment did not utilize the SF-36 or other measures specific to OA because these data were not readily available. It is crucial to interpret these results with caution because the SF-12 examines the general quality of life rather than the quality of life related to OA, even after considering other factors that might influence the overall quality of life in the current analyses. To address these limitations, future research should explore the impact of MMP on HRQOL while taking into account the aforementioned limitations.

## 5. Conclusions

Baseline MMP was associated with a longitudinal decline in the PCS of HRQOL and the KOOS for quality of life among individuals with or at risk of knee OA. Two or more painful sites at baseline were associated with reduced PCS of HRQOL and the KOOS for quality of life. However, no association is observed between the MCS of HRQOL and multisite pain. Having one painful site was associated with a longitudinal decline in the KOOS for quality of life subscale scores. Therefore, strategies in primary healthcare should target MMP assessment and design interventions to preserve and enhance the PCS of HRQOL in individuals with or at risk of OA. For patients with or at risk of knee OA, this means that managing pain at multiple sites may help in maintaining a better quality of life, highlighting the need for personalized treatment plans, regular monitoring, and patient education to encourage early treatment and adherence to pain management strategies.

## Figures and Tables

**Table 1:** Baseline demographics and clinical characteristics.

Factors	No Pain *n* = 198	1 Site Pain *n* = 418	2 Site Pain *n* = 672	Multisite Pain *n* = 3507	*p* *
Age, years (mean ± SD)	61.9 ± 9	62.1 ± 9	61.1 ± 9	61.2 ± 9	0.087
Sex, females, (% within pain groups)	103 (52.0)	202 (48.3)	325 (48.4)	2174 (62.0)	<0.001
Race, White, (% within pain group)	174 (87.9)	360 (86.1)	542 (80.7)	2713 (77.4)	<0.001
BMI, kg/m^2^ (mean ± SD)	26.54 ± 4.3	27.71 ± 4.4	28.50 ± 4.9	28.87 ± 4.9	<0.001
Education, *n*, (% within pain group)					0.002
High school/less	28 (14.1)	45 (10.8)	96 (14.4)	606 (17.4	
Some college/graduate	170 (85.9)	371 (89.2)	571 (85.6)	2867 (82.6)	
Depression (mean ± SD)	3.63 ± 4.0	4.78 ± 5.6	5.40 ± 5.8	7.24 ± 7.4	<0.001
PASE (mean ± SD)	171 ± 82	168 ± 84	164 ± 88	158 ± 81	0.010
Right knee KL grades, (% within pain group)					<0.001
Grade 0	111 (59.4)	164 (41.7	225 (35.8	1190 (36.5)	0.14
Grade 1	29 (15.5)	71 (18.1)	114 (18.2)	569 (17.5)	
Grade 2	42 (22.5)	95 (24.2)	176 (28.0)	922 (28.3)	0.14
Grade 3	4 (2.1)	51 (13.0)	87 (13.9)	467 (14.3)	
Grade 4	1 (0.5)	12 (3.1)	26 (4.1)	112 (3.4)	
Left knee KL grades, (% within pain group)					0.32
Grade 0	104 (55.3)	179 (45.3)	253 (39.9)	1243 (38.1)	<0.001
Grade 1	37 (19.7)	75 (19.0)	104 (16.4)	577 (17.7)	
Grade 2	38 (20.2)	75 (19.0)	154 (24.3)	874 (26.8)	
Grade 3	9 (4.8)	54 (13.7)	95 (15.0)	467 (14.3)	
Grade 4	0 (0)	12 (3.0)	28 (4.4)	104 (3.2)	
Chronic conditions, (% within pain group)					<0.001
No chronic condition	168 (85.3)	335 (81.1)	528 (79.6)	2533 (73.3)	
1 chronic condition	21 (10.7)	45 (10.9)	81 (12.2)	577 (16.7)	
≥2 chronic conditions	8 (4.1)	33 (8.0)	54 (8.1)	346 (10.0)	
					
PCS (mean ± SD)	55.54 ± 6.6	52.84 ± 6.6	51.50 ± 7.2	47.45 ± 9.5	<0.001
MCS (mean ± SD)	55.71 ± 5.3	55.02 ± 6.6	54.83 ± 6.5	53.60 ± 8.1	<0.001
KOOS quality of life (mean ± SD)	94.26 ± 10.53	78.82 ± 21.36	72.97 ± 21.00	62.75 ± 21.85	<0.001

SD: standard deviation. BMI: body mass index. KL: Kellgren Lawrence. PCS: physical component summary. MCS: mental component summary. KOOS: knee injury and OA outcome score. * *p* indicates the *p*-value that was based on the chi-square for the categorical variables or one-way ANOVA for the continuous variables.

**Table 2:** Generalized estimating equations (GEEs) with linear regression for the relationship between baseline multisite pain and overtime physical component summary for quality of life.

Factors	Model (*n* = 4389)		
	B	SE	*p*-Value
No painful site	1.00		
1 painful site	−0.92	0.36	0.011
2 painful sites	−1.94	0.35	<0.001
Multisite pain (≥3 painful sites)	−4.68	0.29	<0.001

Abbreviations: B: Beta (unstandardized coefficients). The model was adjusted for age, sex, race, education, BMI, KL grade, depressive symptoms, physical activity level, KL grades for right and left knees, and chronic conditions.

**Table 3:** GEE with linear regression for the relationship between baseline multisite pain and over-time mental component summary for quality of life.

Factors	Model (*n* = 4389)		
	B	SE	*p*-Value
No painful site	1.00		
1 painful site	−0.20	0.27	0.46
2 painful sites	0.06	0.25	0.79
Multisite pain (≥3 painful sites)	0.21	0.23	0.36

Abbreviations: B: Beta (unstandardized coefficients). The model was adjusted for age, sex, race, education, BMI, KL grade, depressive symptoms, physical activity level, KL grades for right and left knees, and chronic conditions.

**Table 4:** GEE with linear regression for the relationship between baseline multisite pain and over time KOOS for quality of life.

Factors	Model (*n* = 4389)		
	B	SE	*p*-Value
No painful site	1.00		
1 painful site	−6.97	1.05	<0.001
2 painful sites	−9.96	0.93	<0.001
Multisite pain (≥ 3 painful sites)	−17.56	0.81	<0.001

Abbreviations: B: Beta (unstandardized coefficients). The model was adjusted for age, sex, race, education, BMI, KL grade, depressive symptoms, physical activity level, KL grades for right and left knees, and chronic conditions.

## Data Availability

The data associated with the paper are available in the Osteoarthritis Initiative via https://data-archive.nimh.nih.gov/oai/ (accessed on 1 July 2024).
